# Hedgehog costimulation during ischemia-reperfusion injury potentiates cytokine and homing responses of CD4^+^ T cells

**DOI:** 10.3389/fimmu.2023.1248027

**Published:** 2023-10-17

**Authors:** Shaoxun Wang, Guiyu Song, Mahsa Nouri Barkestani, Zuzana Tobiasova, Qianxun Wang, Quan Jiang, Roberto Lopez, Yasmin Adelekan-Kamara, Matthew Fan, Jordan S. Pober, George Tellides, Dan Jane-wit

**Affiliations:** ^1^ Department of Cardiology, West Haven Veterans Affairs (VA) Medical Center, West Haven, CT, United States; ^2^ Section of Cardiovascular Medicine, Yale University School of Medicine, New Haven, CT, United States; ^3^ Department of Surgery, Yale University School of Medicine, New Haven, CT, United States; ^4^ Department of Obstetrics and Gynecology, Shengjing Hospital of China Medical University, Shenyang, China; ^5^ Department of Immunobiology, Yale University School of Medicine, New Haven, CT, United States; ^6^ Yale College, Yale University, New Haven, CT, United States; ^7^ Faculty of Medicine, Imperial College, London, United Kingdom

**Keywords:** ischemia-reperfusion (I/R) injury, alloimmune, T cell, hedgehog, humanized model mouse

## Abstract

**Introduction:**

Ischemia reperfusion injury (IRI) confers worsened outcomes and is an increasing clinical problem in solid organ transplantation. Previously, we identified a “Ptch^Hi^” T-cell subset that selectively received costimulatory signals from endothelial cell-derived Hedgehog (Hh) morphogens to mediate IRI-induced vascular inflammation.

**Methods:**

Here, we used multi-omics approaches and developed a humanized mouse model to resolve functional and migratory heterogeneity within the PtchHi population.

**Results:**

Hh-mediated costimulation induced oligoclonal and polyclonal expansion of clones within the PtchHi population, and we visualized three distinct subsets within inflamed, IRI-treated human skin xenografts exhibiting polyfunctional cytokine responses. One of these PtchHi subsets displayed features resembling recently described T peripheral helper cells, including elaboration of IFN-y and IL-21, expression of ICOS and PD-1, and upregulation of positioning molecules conferring recruitment and retention within peripheral but not lymphoid tissues. Ptch^Hi^ T cells selectively homed to IRI-treated human skin xenografts to cause accelerated allograft loss, and Hh signaling was sufficient for this process to occur.

**Discussion:**

Our studies define functional heterogeneity among a Ptch^Hi^ T-cell population implicated in IRI.

## Background

Ischemia-reperfusion injury (IRI) is a condition where donor tissues subjected to protracted *ex vivo* hypoxia develop complement-mediated damage upon surgical revascularization. Tissues subjected to IRI show poor peri-operative function and have worsened short- and long-term survival (1–5). In an effort to increase organ availability, recent program changes to organ allocation have newly allowed tissues from suboptimal and geographically distant donors to become eligible for transplantation (6–9). *Post-hoc* analyses showed that these newly eligible tissues were susceptible to developing IRI, and the cumulative prevalence of IRI has consequently risen by ~25% (10–13). IRI is now estimated to affect between 40% and 50% of transplant recipients and is an increasing problem in the field of transplantation.

To address this emerging issue, we have investigated how IRI affected the capacity of donor endothelial cells (ECs) to stimulate allogeneic T cells (14–16). During IRI, developmental pathways, including Hedgehog (Hh) signaling become re-activated within injured tissues, ostensibly to promote wound repair (17, 18). Hh signaling supports prenatal organogenesis, and postnatal reactivation of Hh signaling specifies programs for cell proliferation and migration to regulate tissue regeneration (19). Mural cells including ECs, pericytes, smooth muscle cells, and fibroblasts, respond to Hh-mediated growth and positioning signals to promote angiogenesis and wound closure (20–22).

CD4^+^ T cells, while lacking primary cilium, organelles mediating Hh signaling, highly express Hh pathway components, including Ptch, Smo, and Gli effectors, and are capable of both receipt and transmission of Hh inputs, possibly via signals derived from immune synapses (23). Hh signaling encodes programs for positive costimulation (24, 25) to regulate type 1 (14), Th2 (26), Treg (27), and Th17 (28) effector polarization.

Due to increasing rates of IRI, recent studies have explored the effects of exogenous Hh ligands as therapies for mitigating IRI-induced tissue injury. These reports showed that Hh ligands, while enhancing peri-operative tissue function (29–31), paradoxically exacerbated post-operative inflammation and led to accelerated graft loss following IRI (32–35). To explain these paradoxical findings, we hypothesized that Hh programs specifying proliferation and migration and intended for wound repair are pathologically subsumed by allogeneic T cells as a costimulatory signal. Following the acquisition of Hh costimulation, respondent Tmem went on to acquire Hh-related growth and migratory phenotypes to mediate IRI-associated tissue injury.

We previously explored this notion using a humanized mouse model incorporating IRI-treated human coronary artery xenografts (14, 15). We found that soluble and surface-bound Hh ligands, including Sonic, Desert, and Indian Hedgehog, were upregulated by complement membrane attack complexes (MACs) during the reperfusion phase of IRI, and the same Hh ligands delivered costimulatory but not mitogenic signals to memory CD4^+^CD45RO^+^ T cells (Tmem), endowing these cells with heightened effector and migratory responses (14).

Hh signaling upregulates the expression of its own component signaling molecules (19), and exploiting this, we found that the observed functional alterations induced by Hh selectively occurred on Tmem highly expressing Ptch1, the surface receptor for Hh ligands. We found that surface levels of Ptch1, a marker for Hh costimulatory strength, were directly proportional to the ability of these cells to mediate chronic inflammation of IRI-treated human artery xenografts (14).

Frequencies of Ptch^Hi^ T cells were increased in patients with delayed graft function (DGF), a clinical manifestation of IRI in renal transplantation (14), and the Ptch^Hi^ population contained a subset displaying a PD-1^hi^ICOS^hi^ CCR2^+^CCR5^+^CXCR5^−^ surface phenotype (14). This subset exhibited features characteristic of recently described T peripheral helper (T_PH_) cells (35), including expression of ICOS, elaboration of IFN-γ and IL-21, and vigorous homing to inflamed tissues (15). To understand the pathogenic effects of Ptch^Hi^ Tmem, we sought to identify populations within the Ptch^Hi^ subset showing similar effector and homing phenotypes relevant to IRI. To do this, we applied multi-omics approaches and tested the relevance of Ptch^Hi^ subsets using a humanized mouse model. This model incorporated human IRI-treated allogeneic skin and autologous lymphoid tissues to allow interrogation of the effector and migratory properties of subsets contained within Ptch^Hi^ Tmem.

## Materials and methods

### Human CD4+ T-cell isolation

All protocols were approved by the Yale Institutional Review Board (Protocol No. 0601000969). PBMCs were isolated from leukopacks obtained from the Red Cross (Philadelphia, PA) using density centrifugation as described previously and cryopreserved in liquid nitrogen. CD4^+^CD45RO^+^ T cells were isolated from thawed cryovials using magnetic bead separation kits (Miltenyi, Charlestown, MA, USA) with HLA-DR Ab (clone L243, Novus No. NB100-77855) and CD45RA Ab-negative depletion (10 μL per cryovial, eBiosciences, 14-0458-82, San Diego, CA, USA). Where indicated, flat-bottom 96-well microtiter plates were coated with anti-CD3 Ab (1 μg/mL, eBiosciences, No. 16003785) in sterile PBS at 4°C overnight, and the following day, isolated CD4^+^CD45RO^+^ T cells (Tmem) were pretreated with soluble anti-CD28 Ab (1 μg/mL, eBiosciences, No. 16-0281-82) or SAG (15 μM) for 1 h prior to addition to anti-CD3 Ab-coated plates. Tmem were stimulated for 48 h prior to use in downstream applications including TCR deep sequencing, single-cell proteomics, mass cytometry, and passive transfer into humanized mice.

### TCR deep sequencing

For TCR deep sequencing, Tmem were isolated as above and seeded in 96-well flat-bottomed microtiter plates at 50,000 cells/well. Ptch^Hi^ and Ptch^Lo^ Tmem were treated with anti-CD3 Ab and soluble anti-CD28 Ab or SAG were stimulated for 48 h as above, and relevant Ptch^Hi^ and Ptch^Lo^ populations were FACS sorted, homogenized via QiaShredder spin columns (Qiagen, #79656, Hilden, Germany) for 2 min at room temperature, and total DNA was isolated using spin columns (Qiagen). Purified total DNA at 200 ng–1 µg was suspended in nuclease-free H_2_O (30 μL) and submitted for TCR deep sequencing (Adaptive Biotechnologies, Seattle, WA, USA). The CDR3 region of the TCR-α and TCR-β chains were separately amplified in multi-plexed PCR reactions using a panel of proprietary primers.

The analysis results, quality control, and summary statistics of CDR3 sequences (templates) were performed using the online ImmunoSEQ Analyzer portal provided by Adaptive Biotechnologies (http://www.immunoseq.com). All samples were quantified for the absolute total rearrangements, which is the sum of unique detectable CDR3 sequences (or rearrangements) in the TCR-α and TCR-β chains.

### FACS analysis

T-cell surface staining used the following primary antibodies at 1:100 for 1 h at 4°C in PBS containing 3% BSA and 0.01% NaN_3_: CD4^+^ (Biolegend, San Diego, CA, USA), HLA-DR FITC (Biolegend, No. 307604), PD-1 PE (eBiosciences, No. 12-2799-42), CCR2 APC (Biolegend, No. 257208), CXCR5 Pacific Blue (Biolegend, No. 356918), and Ptch1 (Cell Signal, clone C53A3, No. 2468T, Cell Signaling Technology, Danvers, MA, USA). For intracellular staining, T cells were fixed and permeabilized using a FOXP3 Staining Kit according to the manufacturer’s specifications (R&D Systems, Cell Signaling Technology, Danvers, MA, USA). Prior to fixation and permeabilization, cells were stained using the following antibodies at 1:100 for 1 h at 4°C: Gli1 (Cell Signal, clone C68H3, No. 3538T), NLRP3 (Cell Signal, clone DRD8T, No. 15101), and cleaved caspase-1 (Cell Signal, Cat. No. 4199). For cellular staining, antibodies included IFN-γ PE (Biolegend), IL-4 PE/Cy7 (Biolegend, No. 500824), and cleaved caspase-1 (Cell Signaling, clone D57A2, No. 4199). T cells were stimulated for 2 h with PMA/ionomycin prior to the addition of GolgiStop (Becton Dickinson, Franklin Lakes, NJ, USA) for 4 h prior to harvest and FACS analysis as previously described (14, 15). T cells were stained with CFSE (Molecular Probes, Fanklin Lakes, NJ, USA) as previously described (36). T cells were gated and analyzed using an LSR II flow cytometer (Becton Dickinson) and sorted using a FACSAria cell sorter (Becton Dickinson).

### *In situ* hybridization

Transplanted skin and lymph nodes were embedded into an optimal cutting temperature compound (OCT) prior to sectioning. Human CD31 and Sonic Hedgehog were detected using RNA probes Hs-PECAM1-O2 (No. 858151, Advanced Cell Diagnostics, CA, Westborough, MA, USA) and Hs-SHH-C2 (No. 600951-C2 per manufacturer’s specification prior to target amplification by RNAscope Fluorescent Multiplex Reagent Kit (No. 320851, Advanced Cell Diagnostics, CA). Imaging was conducted by laser scanning confocal microscopy using a Leica TCS SP8 Gated STED 3X super-resolution microscope (Leica Microsystems Inc., IL, Wetzlar, Germany). Images were taken at ×400 magnification. Z-stacks were taken at 1 μm apart. A total of 15 Z images were taken, and the Z-projection images were shown. Merged images were deconvoluted into red, green, and, in the case of triple colocalization studies, blue color channels. Pixels ≤1,500 were selected for analysis in the case of Sonic Hedgehog (Shh) and CD31 and sequentially analyzed for colocalization, with colocalized pixels being defined as < 50 pixel diameters.

### Multiplex laser bead assay

Polystyrene beads containing fluorescent dyes were coated with a capture antibody specific to a given protein analyte. Color-coded beads were then analyzed using a bead analyzer (Bio-Plex 200) containing a dual-laser system where the fluorescent dye within each bead is activated and a second laser excites the fluorescent conjugate (streptavidin-PE) that has been bound to the beads during the assay. The amount of conjugate detected by the analyzer is in direct proportion to the amount of the target analyte, which can be quantified using a standard curve (Eve Technologies, Calgary, Canada). Due to wide ranges in quantities among elaborated cytokines, heatmap colors for each row were computed relative to the overall average for that one particular cytokine.

### Single-cell proteomics

Naïve Tmem were stimulated with anti-CD3 (1 μg/mL) in the presence of SAG (1 μM) for 48 h, and cells highly expressing Ptch1 were FACS sorted as “Ptch^Hi^” Tmem. As a comparator, a second set of Tmem stimulated with anti-CD3 in the presence of soluble anti-CD28 (1 μg/mL) for 48 h, and cells showing low expression of Ptch1 from these samples were similarly FACS sorted as “Ptch^Lo^” Tmem. Ptch^Hi^ and Ptch^Lo^ Tmem were subsequently subjected to 32-plex single-cell proteomics (Isoplexis, Branford, CT, USA). Viable T cells were washed and resuspended in complete RPMI media at a density of 1 × 10^6^ cells/mL. Approximately 100 µL of T-cell suspension were seeded into the 96-well flat-bottom plate and stimulated with PMA/ionomycin for 4 h at 37°C. Approximately 30 µL of the cell suspension was loaded into an IsoCode chip containing ~12,000 microchambers prepatterned with a complete copy of a 32-plex antibody array. After 16-h-on-chip incubation at 37°C, protein signals from ~1,000 live single cells were captured and analyzed by fluorescence ELISA-based assay. Cytokine effector responses, including measures of polyfunctionality, were analyzed by IsoSpeak software. Advanced data visualization algorithms, including polyfunctional heatmap, polyfunctional activation topology-principal component analysis (PAT-PCA), and t-SNE were performed using the IsoSpeak software suite.

### Animal studies

Fresh tonsillar and skin tissues were obtained as de-identified samples from the Yale Department of Pathology under IRB-approved protocols approved by the Yale Human Investigations Committee (No. 25173, No. 2000031850). Human tonsillar tissues were obtained from children aged 5–15 years old. Human skin tissues were obtained from adults aged 20–40 years old undergoing mammoplasty. All animal protocols were approved by the Yale Institutional Animal Care and Use Committee (Protocol No.: 2021-20175) and were performed in accordance with institutional guidelines. A portion of fresh, human tonsillar tissue was implanted subcutaneously in the ear of SCID/beige immunodeficient hosts (H-2^d^) lacking T cells and B cells and containing dysfunctional NK cells, and the remainder of the tonsillar tissue from the same host was cryopreserved. Upon notification of availability, fresh human skin tissues were obtained and subdermal fat tissues were removed. To induce IRI, full-thickness skin segments were then placed at 0% FiO_2_ and 5% CO_2_ for 37°C for 8 h in DMEM in organ culture prior to implantation onto the dorsal flanks of SCID/bg hosts containing subcutaneous human LN implants.

For skin transplantation, full-thickness human skin was prepared using a dermatome, grafted via sutures on the dorsal flank of murine hosts, and secured with a bandage for 7 days. For passive transfer, the cryopreserved portions of human LN tissues were placed into single-cell suspension, stimulated with anti-CD3 (1 μg/mL) in the presence of anti-CD28 (1 μg/mL) or SAG (15 μM) for 48 h prior to FACS sorting of relevant Ptch^Hi^ and Ptch^Lo^ populations, labeling with intravital dyes (IVIS 680, PerkinElmer; IVIS 770, PerkinElmer, Waltham, MA, USA), and passive transfer of these cells at 1 × 10^6^ cells per host via jugular vein injection. Passively transferred cells were autologous to implanted human LN and allogeneic to IRI-treated skin grafts. Where indicated, following passive transfer, hosts receive once-daily injections of SAG (5 mg) i.p. for 3 days. Sample sizes for all studies were *n* = 4–5 hosts per group to enable the calculation of standard deviations. Skin xenografts from the same human donor were randomized among treatment groups. The percentage of necrosis area of the skin graft was calculated over time, and ≥80% skin necrosis was operationally used to define rejection (37).

### Mass cytometry (CyTOF) studies

The relevant metal was conjugated to each antibody according to the manufacturer’s specifications (Fluidigm Maxpar, South San Francisco, CA, USA). Antibody–metal pairs are shown in **Table 1**. Following isolation of CD4^+^CD45RO^+^ Tmem via magnetic bead separation, Tmem were resuspended with T-cell media containing 1:10,000 benzonase (25 U/mL), washed, blocked with Fc-receptor blocking solution (Miltenyi), and Ab cocktail was added at a volume of 50 μL to each sample for a total staining volume of 100 μL for 30 mintes at room temperature. Samples were then washed and incubated with 1:500 dilution of 25 cisplatin for a final concentration of 50 μM for 1 min at room temperature. Samples were then washed, fixed, and permeabilized (eBioscience) at 4°C overnight. The next day, Ir-intercalator (Fluidigm) was added at 1:1,000 dilution in 1 mL fixation/permeabilization butter for a final concentration of 125 nM at 4°C overnight. Cells were then washed and resuspended in MilliQ water prior to analysis using the CyTOF Helios instrument (Fluidigm). Metal-conjugated antibodies used in the study are listed in **Table 1**.

**Table 1:** CyTOF antibodies.

Metal	Target	Ab clone
191Ir	DNA	Cell-ID
193Ir	DNA	Cell-ID
103Rh	Live/Dead	Cell-ID
141Pr	CD3	UCHT1
145Nd	CD4	RPA-T4
156Gd	CD183 (CXCR3)	G025H7
173Yb	CD184 (CXCR4)	12G5
154Sm	CD185 (CXCR5)	J252D4
160Gd	CXCR6 (CD186)	K041E5
153Eu	CD192 (CCR2)	K036C2
175Lu	CD193 (CCR3)	5E8
158Gd	CD194/CCR4	L291H4
171Yb	CD195 (CCR5)	NP-6G4
176Yb	CD196 (CCR6)	G034E3
167Er	CD197 (CCR7)	G043H7
165Ho	IFNg	B27
172Yb	IL-21	3A3-N2
144Nd	IL-4	MP4-25D2
150Nd	IL-22	22URTI
169Tm	IL-17A	BL168
163Dy	BCL-6	K112-91
155Gd	CD279 (PD-1)	EH12.2H7
106Cd	CXCR1	427.5
110Cd	CXCR2	48311
195Pt	CXCR7	11G8
114Cd	CCR1	53504
111Cd	CCR8	191704
168Er	CD199 (CCR9)	L053E8
116Cd	CCR10	314305R
146Nd	PITPNM3	Polyclonal
147Sm	CCRL2	152211
152Sm	XCR1	Polyclonal
161Dy	ACKR1/Duffy	358307
162Dy	CCRL1/CCR11/ACKR4	674144
164Dy	S1PR5	282503
170Er	Ptch1	C53A3
194Pt	BLIMP1	3H2-E8
166Er	IL-2	MQ1-17H12
151Eu	CD103	Ber-ACT8
196Pt	Cleaved caspase-1	14F468
148Nd	CD278/ICOS	C398.4A

### Statistical methods

TCR repertoire richness was determined by applying a clonality score, adapted from the Gini–Simpson index, calculated as the square root of the Simpson diversity metric based on productive CDR3 rearrangements. Simpson’s clonality was calculated as the square root of the sum of all observed rearrangements over the square fractional abundances of each rearrangement and was used due to its robustness across differences in sampling depths. Simpson’s clonality values range between 0 and 1, with values approaching 1 representing a monoclonal population. Clone distribution slopes were calculated as the slope of the best-fit line on a log–log plot comparing the range of clonal frequencies to the number of clones at each frequency as previously described (38). An increased clone distribution slope represents increased populational diversity as more rare clones become expanded. We assessed the differences in total rearrangements and clonality among different groups using the nonparametric Wilcoxon signed-rank tests. Comparisons between groups in mass cytometric analyses were performed using a two-sample Student’s *t*-test using Origin computer software. Standard deviations are reported throughout the text.

### Data availability

All data and methods are available from the authors upon reasonable request. The following transcriptomic datasets were retrieved from the Gene Expression Omnibus: GSE147089 (*n* = 224) [https://www.ncbi.nlm.nih.gov/geo/query/acc.cgi?acc=GSE147089], GSE112943 (*n* = 21) [https://www.ncbi.nlm.nih.gov/geo/query/acc.cgi?acc=GSE112943], and GSE97779 (*n* = 23) [https://www.ncbi.nlm.nih.gov/geo/query/acc.cgi?acc=GSE97779]. TCR deep sequencing data are available at https://clients.adaptivebiotech.com/pub/wang-2023-fi. All data are available from the authors upon reasonable request.

## Results

### Hh costimulation elicits oligoclonal and polyclonal expansion of Tmem

Human ECs, unlike rodent ECs, express costimulatory molecules including ICOS-L and LFA-3 to enable direct allorecognition, a process where ECs act as antigen-presenting cells (APCs) to directly prime CD4^+^CD45RO^+^ memory T cells (Tmem). This process may modulate both the growth and migratory responses of cognate Tmem.

We previously found that human ECs produce Hh ligands *in vitro and in vivo (* 14) following IRI and that these Hh ligands acted as costimulatory signals to a defined T-cell subset highly expressing Ptch1, a population we termed Ptch^Hi^ Tmem (14). While producing Hh ligands as costimulators, ECs lack CD80/86, costimulatory ligands for CD28 expressed on Tmem. In contrast, professional APCs like dendritic cells express CD80/86 and traffic to draining lymph nodes to present EC-derived alloantigens to cognate Tmem (39). To model the effects of these distinct routes of costimulation *in vitro*, we exposed αCD3 Ab-treated Tmem to smoothened agonist (SAG) to mimic the effects of Hh ligands, all three of which were induced by MACs in IRI-treated ECs (14); and as controls we exposed αCD3 Ab-treated Tmem to αCD28 Ab to model APC : Tmem interactions in draining LNs.

**Figure 1:**
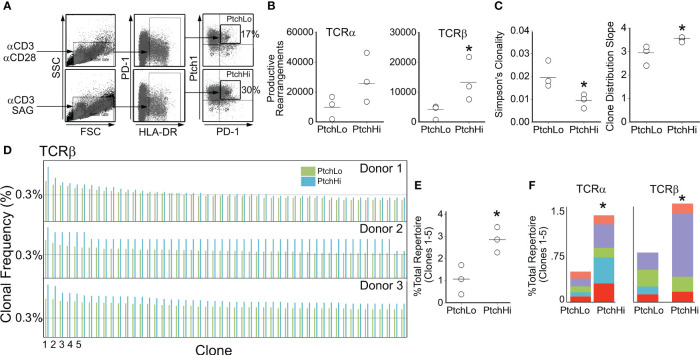
Hh costimulation elicits oligoclonal and polyclonal expansion of Tmem. Human CD4^+^CD45RO^+^ T cells (Tmem) were stimulated with plate-bound αCD3 Ab (1 μg/mL) in the presence of soluble αCD28 (1 μg/mL) or SAG (15 μM) for 48 h to generate Ptch^Lo^ and Ptch^Hi^ Tmem, respectively **(A)**. CDR3 analysis of TCR-α and TCR-β chains showed increased clonal diversity in Ptch^Hi^ Tmem compared to Ptch^Lo^ Tmem **(B, C)**. Ptch^Hi^ Tmem showed oligoclonal and polyclonal expansion **(D–F)**. **(A–F)** *n* = 3 donors tested. ^*^ *p* < 0.05. For **(B, C, E)**, Student’s *t-*test was used for statistical comparisons.

To gain insight into the effects of Hh costimulation on Ptch^Hi^ T cells, we initially examined the Ptch^Hi^ repertoire via deep sequencing of the TCR-α and TCR-β chains. Because we postulate that Hh costimulation occurs within tissues, we focused on Tmem from peripheral blood, which likely forms interchangeable pools with populations recruited to tissues. In contrast to certain autoimmune conditions where one or a few HLA-restricted driver clones dictate the populational response, alloimmune responses against EC-derived HLA molecules are highly polyclonal. To model these effects, we polyclonally stimulated Tmem from three separate donors with plate-bound αCD3 in the presence of either SAG or αCD28 Ab. Following activation, we sorted the respective populations shown in **Figure 1A**, which we termed Ptch^Hi^ and Ptch^Lo^ Tmem, respectively, and performed unbiased sequencing of the TCR-α and TCR-β chains.

Ptch^Hi^ Tmem highly coexpressed PD-1 and showed increased expression of both surface Ptch1 and PD-1 (**Supplementary Figure S1A**) in alignment with our prior findings (14). We call the T-cell population expanded by SAG, “Ptch^Hi^” Tmem, and we call its comparator subset generated in the presence of αCD28 and showing lower Ptch1 expression, “Ptch^Lo^” Tmem. General survey statistics are shown in **Table 2**. Among three separate donors, we detected a total of 19,462 productive rearrangements involving the TCR-α chain and 52,707 productive rearrangements involving the TCR-β chain. Hh costimulation significantly increased the number of productive TCR-β rearrangements (**Figure 1B**). The most frequently utilized J isoforms in both the TCR-α and TCR-β chains were not significantly altered by Hh costimulation (**Supplementary Figure S1B**); however, usage of certain low-frequency J isoforms was significantly increased by Hh (**Supplementary Figure S1C**). CDR3 lengths showed Gaussian distributions in all groups examined, and CDR3 lengths were unchanged in Ptch^Hi^ vs. Ptch^Lo^ Tmem (**Supplementary Figure S1D**). The clonal repertoire of Ptch^Lo^ vs. Ptch^Hi^ showed comparatively higher repertoire similarity according to Simpson’s clonality metric within a given donor than between donors (**Supplementary Figure S1E**).

**Table 2:** General TCR survey statistics.

Cell type	Donor	TCR	Unique rearrangements	Productive rearrangements	%Productive rearrangements
PtchLo	1	α	19,851	11,674.37	58.81%
		β	6,846	5,439.15	79.45%
	2	α	27,699	16,672.03	60.19%
		β	6,348	5,071.42	79.89%
	3	α	3,509	1,815.21	51.73%
		β	941	760.52	80.82%
PtchHi	1	α	23,201	13,451.94	57.98%
		β	8,088	6,476.06	80.07%
	2	α	79,351	46,317.18	58.37%
		β	28,875	23,036.48	79.78%
	3	α	45,942	26,843.91	58.43%
		β	15,037	11,924.34	79.30%

As above, Tmem subjected to Hh costimulation showed an increased number of productive rearrangements (**Figure 1B**), and clonal analyses using two separate metrics previously applied to alloresponses (38) revealed that Ptch^Hi^ Tmem showed increased repertoire diversity involving both TCR-α and TCR-β (**Figure 1C**). In frequency analyses, we observed that Hh costimulation had induced a disproportionate, oligoclonal expansion of two to five of the highest-frequency clones within each donor while polyclonally expanding 347–365 lower-frequency clones. To illustrate this, the top 50 rearrangements for each donor for TCR-β are shown in **Figure 1D**. The top 5 highest frequency clones, when analyzed in combination (**Figure 1E**) or separately (**Figure 1F**), for TCR-α and TCR-β were significantly expanded by Hh costimulation. The highest expanded clones in each donor whose sequences are shown in **Supplementary Figure S1E** showed low inter-donor CDR3 sequence similarity as measured by Morista indices (**Supplementary Figure S1F**). This feature of low inter-donor similarity was recapitulated at a repertoire level (**Supplementary Figure S1G**). The polyclonally expanded repertoire among all three donors did, however, contain shared clones in Ptch^Hi^ populations, and these clones were universally of low frequency (**Supplementary Figure S1H**). Hh costimulation diversifies the TCR repertoire of Ptch^Hi^ Tmem by inducing both oligoclonal and polyclonal expansion of respondent clones.

### Hh costimulation elicits polyfunctional cytokine responses in Ptch^Hi^ Tmem

Hh costimulation diversified the TCR repertoire, and we tested how this may have affected the cytokine effector response. To test this, we performed multiplexed laser bead assay in conjunction with single-cell proteomics via bead-based capture of barcoded Ptch^Hi^ vs. Ptch^Lo^ Tmem. These dual approaches were employed to concurrently obtain information regarding quantities of inflammatory mediators as well as frequencies of cells producing such mediators. Ptch^Hi^ and Ptch^Lo^ Tmem were generated under nonpolarizing conditions by plate-bound αCD3 with concurrent exposure to SAG or αCD28. Following this, culture supernatants were analyzed via a multiplexed laser bead assay, and cells were subjected to single-cell proteomics.

**Figure 2:**
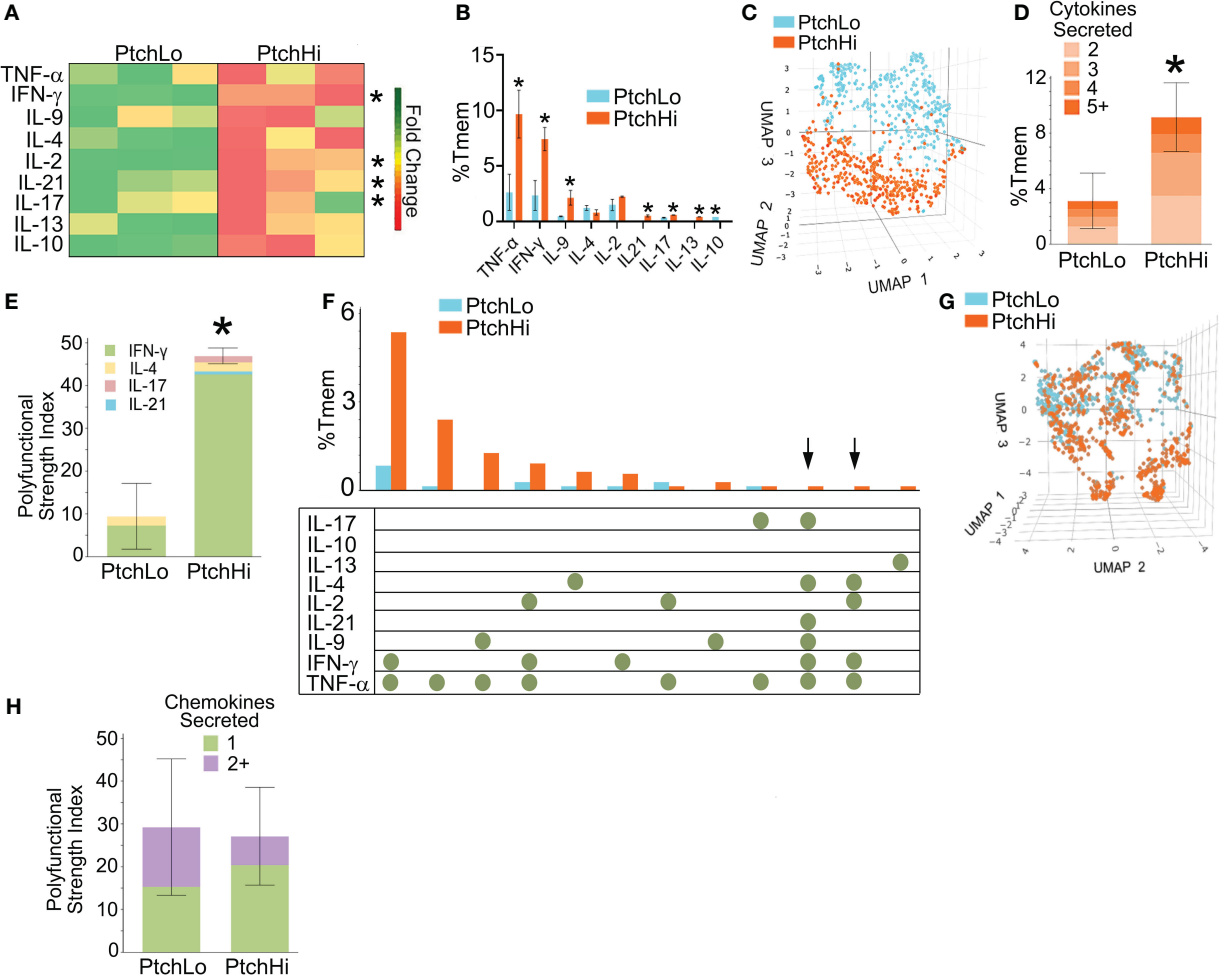
Hh costimulation elicits polyfunctional cytokine responses in Ptch^Hi^ Tmem. Culture supernatants from Ptch^Lo^ and Ptch^Hi^ Tmem were analyzed using a multiplexed laser bead assay **(A)**. Single-cell proteomics showed quantitative **(B)** and qualitative **(C)** differences in the frequencies of the effector Ptch^Lo^ and Ptch^Hi^ Tmem. Compared to Ptch^Lo^ Tmem, PtchHi Tmem showed polyfunctional cytokine responses **(D)** that were strongly driven by IFN-γ **(E)**. Subset frequencies based on cytokine function among Ptch^Lo^ and Ptch^Hi^ Tmem **(F)**. Chemokine responses in Ptch^Hi^ vs. Ptch^Lo^ Tmem did not show qualitative differences **(G)** or changes in polyfunctionality **(H)**. In **(A)**, heatmap colors for each row were computed relative to the overall average for that particular cytokine. *n* = 3 separate donors tested. ^*^ *p* < 0.05, *** *p* < 0.001. For **(D, E, G, H)**, Student’s *t-*test was used for statistical comparisons.

We found that Hh costimulation significantly potentiated the elaboration of cytokine mediators involved in IRI (**Figure 2A**). Due to the wide range in quantities of elaborated cytokines, heatmap colors for each row were computed relative to the overall average for that one particular cytokine, and raw values are shown in **Table 3**. Frequencies of cytokine-elaborating clones were similarly increased for many inflammatory mediators (**Figure 2B**). We tested correlations among cytokine levels and corresponding frequencies of cytokine-producing cells. Among the cytokines analyzed, cytokines implicated in IRI-mediated tissue pathology, including IFN-γ, IL-17, and IL-21, were significantly elevated in both multiplexed laser bead and single-cell proteomic assays. In UMAP plots, Ptch^Hi^ and Ptch^Lo^ populations showed remarkably distinct effector profiles (**Figure 2C**). We segregated analyses of effector function based on cytokines vs. chemokines to ask which of these responses drove the divergent effector responses. In this analysis, we found that the frequencies of polyfunctional clones elaborating ≥2 cytokines were significantly enhanced by Hh costimulation (**Figure 2D**). Among polyfunctional clones elaborating cytokines defining Th2, Th9, and Th17 subsets, we observed a marked increase in clones elaborating IFN-γ, a hallmark of the type 1 response that predominates in IRI (**Figure 2E**). The heightened type 1 response induced by Hh costimulation aligned with our prior observations (14, 15) and may have occurred due to the potentiated release of cytokines like IL-9 and IL-21, whose increased levels promote T-Bet. Combinatorial frequencies of effector clones among Ptch^Lo^ and Ptch^Hi^ populations are shown in **Figure 2F**. Many polyfunctional subsets were expanded by Hh costimulation, and in particular, we noted two populations that were present in Ptch^Hi^ but not Ptch^Lo^ Tmem and that were highly polyfunctional, elaborating ≥4 cytokines each (arrows). In these analyses, cytokine-producing cells below the pre-defined threshold for detection for the assay are listed in **Table 4**. In a prior report (14), we previously tested the effects of dual Hh costimulation plus CD28 costimulation, which might occur in instances where tissue-infiltrating Tmem become activated via indirect allorecognition involving professional APCs positioned near IRI-treated ECs. In these prior studies, dual costimulation with αCD28 Ab plus SAG phenocopied Hh costimulation alone, and respondent HLA-DR^+^ Tmem highly elaborated IFN-γ (14).

**Table 3:** Cytokine concentration (pg/mL).

	Ptch^Lo^			Ptch^Hi^		
**TNF-α**	666.56	211.11	1,439.99	2,704.02	1,153.04	2,411.80
**IFN-γ**	398	407	297.00	3,475.00	3,462.00	4,506.00
**IL-9**	43.00	58.00	50.00	84.00	86.00	49.97
**IL-4**	14.78	8.83	7.71	402.34	37.01	414.66
**IL-2**	9.30	7.80	9.80	504.00	288.76	259.01
**IL-21**	2.12	5.39	6.97	44.15	31.34	14.46
**IL-17A**	5.99	149.92	225.87	1,516.14	704.24	26.70
**IL-13**	1,950.00	100.00	42.00	7,517.25	3,838.44	3,029.69
**IL-10**	40.27	79.66	130.42	7,719.95	9,031.78	1,224.84

**Table 4:** Undetected mediators.

IL-1β	
IL-5	
IL-6	
IL-7	
IL-8	
IL-15	
MCP-1	
MCP-4	
TGF-β	

We performed the same analysis based on chemokine levels and frequencies of chemokine-expressing clones in Ptch^Hi^ Tmem. While a majority of Ptch^Hi^ clones elaborated type 1-dependent chemokines, including MIP-1α, Ptch^Hi^ Tmem did not show qualitatively distinct chemokine responses compared to Ptch^Lo^ following Hh costimulation (**Figure 2G**). Moreover, in contrast to cytokine effectors, Hh costimulation did not generate polyfunctional chemokine responses (**Figure 2H**). These data indicated that the differential effector response among Ptch^Lo^ vs. Ptch^Hi^ Tmem in **Figure 2C** was principally driven by a diversified cytokine effector profile in Ptch^Hi^ Tmem. Hh costimulation diversifies the cytokine response of Ptch^Hi^ Tmem, generating subsets displaying heightened and polyfunctional responses, particularly those involving IFN-γ.

### Polyfunctional Ptch^Hi^ subsets coexpress molecules for peripheral tissue homing

We have previously utilized a human artery xenograft model to examine EC-mediated direct allorecognition, a response unique to human ECs. Using this model, we found that human artery xenografts subjected to IRI showed enhanced Tmem recruitment to an expanded neointima *in vivo* (14, 15). To define subset(s) recruited to IRI-treated tissues, we examined spatial positioning molecules that were expressed on polyfunctional Ptch^Hi^ Tmem. To do this, we performed multi-parameter analyses of surface chemokine receptors, integrins, and lipid receptors using mass cytometry.

**Figure 3:**
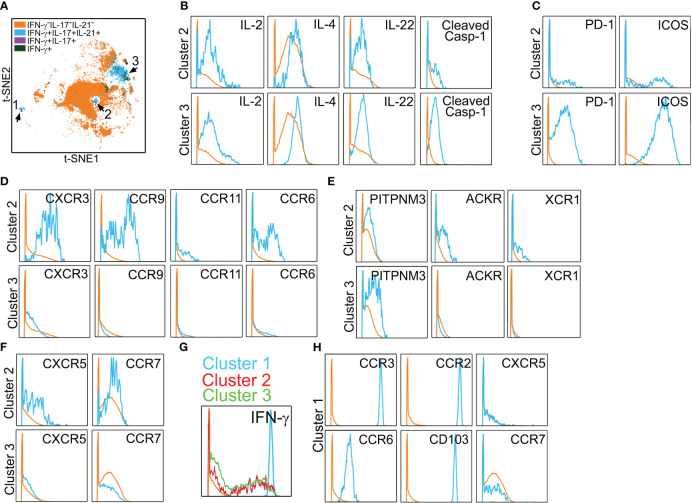
Polyfunctional Ptch^Hi^ subsets coexpress molecules for peripheral tissue homing. Mass cytometry of Ptch^Hi^ Tmem was performed, and a concatenated t-SNE plot of Ptch^Hi^ Tmem from three separate donors uncovered three effector subsets **(A)**. Effector subsets within clusters 2 and 3 show polyfunctional cytokine responses **(B)**. Cluster 3 highly coexpresses costimulatory molecules **(C)**, while cluster 2 expresses a broad array of classical **(D)** and atypical **(E)** chemokine receptors for peripheral tissue homing as well as chemokine receptors for homing to lymphoid tissues **(F)**. Relative to clusters 2 and 3, cluster 1 shows strong IFN-γ responses **(G)** and uniquely expresses molecules for positioning and retention within peripheral but not lymphoid tissues **(H)**.

In initial studies performed *in vitro* to allow optimization, Ptch^Hi^ Tmem was stained for a total of 38 molecules via mass cytometry (**Table 1**). Following the identification of viable, CD3^+^CD4^+^Ptch1^+^PD-1^+^ singlets, we gated on HLA-DR^+^ subsets that produced IFN-γ, IL-17, and IL-21, cytokines appearing in the multiplexed laser bead and single-cell proteomic assays above and that have been implicated in IRI. This gating strategy revealed three distinct subsets, clusters 1–3 (blue, **Figure 3A**). In addition to the cytokines above, clusters 2 and 3 additionally elaborated IL-2, IL-4, and IL-22 and showed cleaved caspase-1 whose activity was previously detected in Ptch^Hi^ Tmem to allow these cells to elaborate IL-1β (**Figure 3B**) (14). Relative to Tmem lacking cytokine elaboration (orange), Ptch^Hi^ cells in clusters 2 and 3 (blue) highly coexpressed costimulatory molecules, including PD-1 and ICOS (**Figure 3C**). Tmem within cluster 2 (blue) broadly expressed canonical chemokine receptors conferring homing to the gut (CCR9), skin (CCR6), and inflamed tissues (CXCR3, CCR11, **Figure 3D**); atypical chemokine receptors (ACKR, XCR4, **Figure 3E**); and positioning molecules allowing homing to lymphoid organs, including CXCR5 and CCR7 (**Figure 3F**).

In t-SNE plots (**Figure 3A**), cluster 1 was more spatially separated from clusters 2 and 3, and Tmem in cluster 1 showed very strong IFN-γ responses (**Figure 3G**). Cluster 1 (blue) Tmem uniquely and strongly coexpressed molecules for homing to inflamed (CCR2, CCR3, CCR6, CD103) but not lymphoid tissues (CXCR5, CCR7, **Figure 3H**) relative to Tmem that did not elaborate cytokines (orange). These data showed that Hh costimulation generates polyfunctional T-cell subsets expressing costimulatory molecules and a diversified pattern of spatial positioning molecules.

### A humanized mouse model examining effector and migratory responses of Ptch^Hi^ Tmem

**Figure 4:**
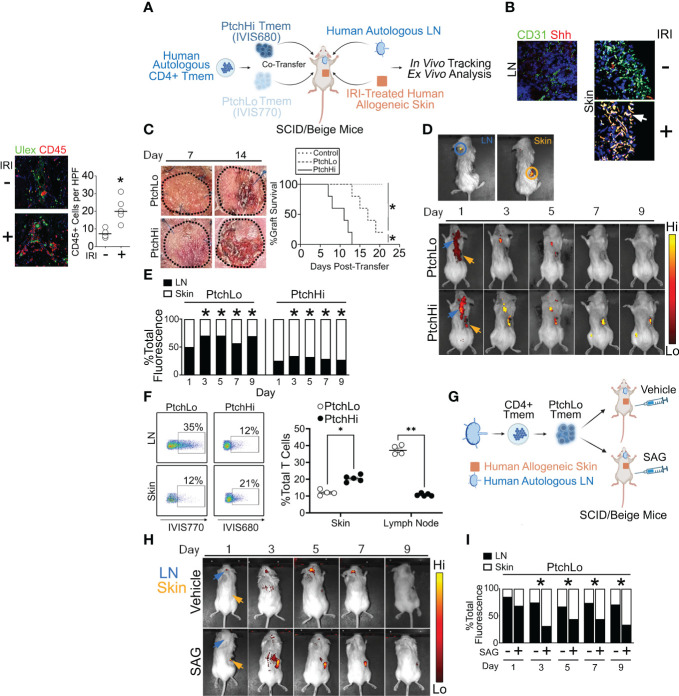
A humanized mouse model examining effector and migratory responses of Ptch^Hi^ Tmem. Human LN and IRI-treated human skin were implanted into immunodeficient SCID/beige mice that passively received Ptch^Hi^ and/or Ptch^Lo^ Tmem that was autologous to LN tissues and allogeneic to skin tissues **(A)**. At the time of passive transfer, Shh transcripts colocalized with CD31^+^ vessels in IRI-treated human skin but not in LN **(B)**. Hosts receiving Ptch^Hi^ Tmem only showed significantly accelerated rejection of allogeneic skin xenografts compared to hosts receiving Ptch^Lo^ Tmem only or hosts receiving no Tmem **(C)**. Ptch^Lo^ and Ptch^Hi^ Tmem were differentially labeled with fluorescent membrane dyes, mixed at 1:1 ratios for a total of 2 × 10^6^ cells, and passively cotransferred into SCID/beige mice bearing human LN and IRI-treated human skin. Ptch^Lo^ and Ptch^Hi^ Tmem migration was tracked *in vivo* over time **(D)**. Fractions of bioluminescence in ROIs encompassing LN and skin tissue were calculated over time and statistically compared with respective bioluminescence values at Day 1 post-transfer **(E)**. Percentages of dye-labeled Ptch^Lo^ and Ptch^Hi^ Tmem were calculated in LN and skin as a percentage of CD3^+^CD4^+^ T cells at Day 5 post-transfer **(F)**. Hosts bearing LN and IRI-treated skin and passively receiving Ptch^Lo^ Tmem were subsequently injected i.p. with vehicle or SAG (5 mg) for 3 days while undergoing IVIS imaging **(G, H)**. Percentages of dye-labeled cells were calculated at each time point post-transfer (*n* = 3 per timepoint per group **(I)**. ^*^ *p* < 0.05, **p < 0.01. For **(C)**, Wilcoxon’s rank sum test was used for statistical comparisons. For **(E, F)**, Student’s *t-*test was used for statistical comparisons.

We subsequently developed a humanized mouse model to test the effector and migratory properties of the Ptch^Hi^ Tmem subsets visualized above. In this model, fresh, human tonsillar LNs were implanted as subcutaneous xenografts in proximity to the ear of immunodeficient SCID/beige mice lacking T and B cells, and a portion of the same LN tissues were placed into single-cell suspension and cryopreserved (**Figure 4A**). Subsequently, human skin that was allogeneic to the implanted tonsillar LN was placed in organ culture and subjected to anoxia, a condition we found was necessary to mimic IRI because skin tissues were of sufficient thickness to be well oxygenated by diffusion. Following this treatment, human skin tissues were placed as full-thickness xenografts on the dorsal flank of SCID/bg hosts. Following implantation of IRI-treated skin, the cryopreserved, tonsillar LN cells above were thawed and used to generate Ptch^Hi^ or Ptch^Lo^ cells *in vitro* via polyclonal stimulation with plate-bound αCD3 Ab in the presence of Hh- or CD28-mediated costimulation. Ptch^Hi^ and Ptch^Lo^ cells generated in this fashion were differentially labeled with fluorescent dyes, IVIS770 and IVIS 680, respectively, prior to being passively transferred either separately or cotransferred at a 1:1 ratio as indicated into SCID/bg hosts via jugular vein injection. The differentially labeled Ptch^Hi^ and/or Ptch^Lo^ cells were autologous to the implanted LNs and allogeneic to the IRI-treated skin tissues. At various times following the transfer, differentially labeled Tmem contained in peripheral skin tissues and/or LN tissues were imaged *in vivo* using bioluminescence and recovered for *ex vivo* analysis.

Female SCID/beige hosts were used as recipients for human tissues due to their higher post-surgery survival compared to males. We used human skin tissues due to their heightened immunogenicity relative to kidney or liver xenografts, thereby permitting discrimination of the effector and migratory properties among Ptch^Hi^ and Ptch^Lo^ cells. Additionally, the large surface area of these tissues enabled the recovery of infiltrating cells, a feature that was lacking in human artery xenografts, which we employed previously (14, 15). During the course of optimization, we found that human LN implanted subcapsularly within mouse kidneys showed high rates of engraftment but failed to induce significant T-cell recruitment, precluding their further use. Various anatomical sites for LN implantation were also tested in pilot studies, including the subcapsular subcapsular liver and subcutaneous sites including the dorsal flank and ear. Tissues subjacent to the ear in mice are highly vascularized, and ear tissues are widely examined as readouts for hapten-induced hypersensitivity. Among the tested sites, subcutaneous skin implantation proximal to the ear allowed the highest rates of engraftment.

Compatible with our prior findings (14), Hh costimulation strongly modulated both T-cell effector function (**Figure 2**) and spatial positioning molecules (**Figure 3**). Due to these strong effects, we tested anatomical sites for Hh ligand production *in vivo*. We performed multiplexed *in situ* hybridization and found that LNs surgically implanted in the ear showed low levels of Sonic Hedgehog (Shh) as did freshly obtained human skin prior to IRI. In contrast, skin tissues subjected to IRI were diffusely stained with Shh in both the epidermis and dermis and heavily colocalized with transcripts for CD31, a marker of ECs (**Figure 4B**). These data aligned with our prior results showing Shh production by ECs in human renal tissues from DGF patients in IRI-treated human artery xenografts (14). ECs within IRI-treated human skin are a source of Hh ligands in our humanized mouse model.

In our initial studies, we examined the effects of IRI on the survival of allogeneic skin grafts. Ptch^Lo^ and Ptch^Hi^ Tmem were cotransferred at 1:1 ratios into mice bearing allogeneic skin grafts subjected either to anoxia to simulate IRI or to normoxia as controls prior to surgical implantation (*n* = 5 per group). As expected, we found that skin grafts subjected to IRI showed accelerated rejection defined as ≥80% scab formation compared to normoxia-treated skin grafts (**Supplementary Figure S2A**). Upon analysis at post-transfer day 14, IRI-treated grafts showed increased immune cell infiltrates (**Supplementary Figure S2B**) and significantly increased epidermal thickening (**Supplementary Figure S2C**), a skin pathology induced by IFN-γ. In alignment with our prior model using human artery xenografts (14), IRI significantly increased Tmem tissue infiltration.

We next assessed the effects of Ptch^Lo^ and Ptch^Hi^ Tmem on the survival of allogeneic skin grafts. Hosts bearing autologous LN and IRI-treated allogeneic skin tissues passively received Ptch^Lo^ Tmem, Ptch^Hi^ Tmem, or no Tmem, and skin rejection was assessed over time (*n* = 5 per group, **Figure 4C**). Compatible with prior results using IRI-treated human artery xenografts (14) and in accord with the data in **Figures 2**, **3**, showing heightened and polyfunctional cytokine responses, hosts receiving Ptch^Hi^ Tmem showed significantly increased perivascular (Ulex) CD45^+^ Tmem infiltration at day 7 (**Supplementary Figure S2D**) and developed significantly accelerated allograft rejection compared to Ptch^Lo^ Tmem (**Figure 4C**). In contrast to the above, hosts receiving no Tmem did not show rejection during the monitoring period.

We next examined the respective migratory behavior of Ptch^Hi^ vs. Ptch^Lo^ Tmem. Ptch^Hi^ and Ptch^Lo^ Tmem were isolated by FACS sorting; differentially labeled using IVIS770 and IVIS680 dyes, respectively; and passively cotransferred at 1:1 ratios into SCID/beige hosts bearing autologous LN and IRI-treated allogeneic skin. Following passive transfer, we were able to use *ex vivo* bioluminescence to spatially track Ptch^Hi^ and Ptch^Lo^ cells in the same host. Regions of interest (ROIs) were drawn around sites of autologous human LN (blue circle) and allogeneic human skin (orange circle), and bioluminescent intensities were calculated within these ROIs.

At post-transfer day 1, we observed bioluminescence signals for both Ptch^Lo^ and Ptch^Hi^ Tmem in regions outside implanted human tissues. This signal may have reflected cells in the hematogenous and/or lymphatic circulation (**Figures 4D, E**). By day 3, bioluminescence signals for both Ptch^Hi^ and Ptch^Lo^ Tmem became more restricted to implanted tissues, enabling analyses of bioluminescence ratios between tissue LN and skin tissues. At this time, Ptch^Lo^ Tmem showed increased LN:skin ratios of bioluminescence values, while Ptch^Hi^ showed significantly reduced LN:skin bioluminescence, and this remarkably divergent pattern was maintained up to ~10–12 days post-transfer, after which time fluorescent labels became too weak to permit reliable *ex vivo* imaging. We obtained similar, divergent recruitment of Ptch^Hi^ vs. Ptch^Lo^ Tmem has similar kinetics when using human splenic tissues in lieu of human LNs (**Supplementary Figure S2E**), indicating that cellular transit between LN and skin tissues likely reflected hematogenous and not lymphatic trafficking.

Dye-labeled Tmem were recovered from LN and IRI-treated skin tissues at day 5 and analyzed by flow cytometry. Supporting bioluminescence findings, in IRI-treated skin tissues, Ptch^Hi^ Tmem were detected at approximately twofold increased frequencies compared to Ptch^Lo^ Tmem (**Figure 4F**). In contrast, autologous LN tissues showed an approximately fourfold comparative enrichment of Ptch^Lo^ Tmem compared to Ptch^Hi^ Tmem. These differential trafficking phenotypes were reproducibly observed across multiple murine hosts. These data showed that Ptch^Hi^ and Ptch^Lo^ Tmem show divergent homing to peripheral vs. lymphoid tissues *in vivo.*

To test whether the differential migratory capacity of Ptch^Lo^ vs. Ptch^Hi^ Tmem was mediated by Hh costimulation, we passively transferred Ptch^Lo^ Tmem receiving αCD28 Ab costimulation into hosts bearing IRI-treated human skin and autologous LN who then received daily treatments of the vehicle or SAG (**Figure 4G**). Compared to vehicle-treated hosts where Ptch^Lo^ Tmem primarily homed to autologous LN, SAG-treated hosts showed increased migration of Ptch^Lo^ Tmem to skin tissues, phenocopying the migratory responses of Ptch^Hi^ Tmem (**Figure 4H**). At each time point, ratios of dye-labeled Tmem were calculated in LN and skin tissues. We found that Hh costimulation significantly increased the ratios of Ptch^Lo^ Tmem homing to skin vs. LN starting at Day 3, and these responses were durable up to day 9 post-transfer (**Figure 4I**, *n* = 3 per timepoint per group). In Ptch^Lo^ Tmem receiving antecedent costimulation with αCD28 Ab, Hh costimulation was sufficient for inducing migratory homing to IRI-treated peripheral tissues.

### Polyfunctional Ptch^Hi^ subsets preferentially home to peripheral tissues *in vivo*

**Figure 5:**
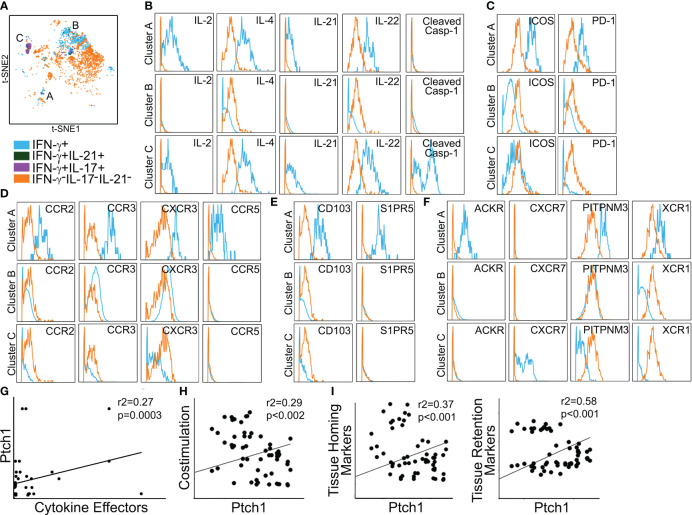
Polyfunctional Ptch^Hi^ subsets preferentially home to peripheral tissues *in vivo*. Ptch^Hi^ Tmem recovered from IRI-treated human skin 5 days post-transfer were analyzed *ex vivo* via mass cytometry. Concatenated t-SNE plots from *n* = 3 separate donors identified three effector subsets **(A)**. Cluster A coexpresses effector cytokines **(B)**, costimulatory markers **(C)**, and chemokine receptors **(D)** compatible with T peripheral helper cells. Cluster A expresses markers for peripheral tissue retention **(E)** and atypical chemokine receptors **(F)**. Correlation analyses were performed on three public RNA seq datasets involving (*n* = 268 patients) as specified in the Materials and methods section. Transcript abundance of Ptch1 showed significant correlations with an index of effector cytokines (**G**, IFN-G, IL-17, IL-21); costimulatory molecules (**H**, ICOS, PDCD1); as well as tissue homing (**I**, left, CCR2, CCR4) and tissue retention (**I**, right, CD103, S1PR5) molecules.

We used mass cytometry to examine Ptch^Hi^ Tmem recovered from IRI-treated skin tissues. Five days post-transfer, Ptch^Hi^ Tmem elaborating IFN-γ, IL-21, and IL-17 were gated and analyzed for spatial positioning molecules. This gating strategy, as before, revealed three distinct effector populations, which may have been derived from clusters 1–3 visualized prior to transfer (**Figure 3**). We call these Ptch^Hi^ Tmem clusters, clusters A–C (**Figure 5A**). Among these clusters, clusters A and C showed highly polyfunctional responses, elaborating IL-2, IL-4, IL-2, IL-22, and cluster C highly expressed cleaved caspase-1 (**Figure 5B**). Cluster A highly coexpressed PD-1 and ICOS (**Figure 5C**), as well as chemokine receptors for peripheral tissue homing (**Figure 5D**; **Supplementary Figure S3A**).

Prior to passive transfer, we detected a subset of Ptch^Hi^ Tmem (cluster 1) that concurrently expressed molecules for both peripheral (CCR2, CCR3, CCR6, CD103) and lymphoid (CXCR5) homing. In contrast to this cluster visualized prior to passive transfer, Tmem in cluster A recovered from IRI-treated skin showed downregulated expression of CXCR5 (**Supplementary Figure S3B**). The effector, costimulatory, and chemokine receptor profile of cluster A was compatible with recently described T peripheral helper cells (T_PH_ cells) (35). In support of this notion, among the three effector clusters, cluster A showed the lowest bcl6:BLIMP1 ratios (**Supplementary Figure S3C**). In addition to the markers above, cluster A was uniquely labeled by molecules allowing tissue retention (CD103, S1PR5, **Figure 5E**) and atypical chemokine receptors (XCR1, PITPNM3, ACKR, **Figure 5F**). Thus, IRI is a complement-mediated condition, and to test the relevance of our findings, we used public RNA seq datasets and performed correlations of effector cytokines, costimulatory molecules, and spatial positioning molecules with Ptch1, the salient marker for Ptch^Hi^ Tmem. We analyzed datasets from three separate conditions involving complement-mediated tissue injury, including antibody-mediated rejection, a complication of DGF; rheumatoid arthritis; and systemic lupus erythematosus. Pearson’s *r* ^2^ showed low–moderate and moderate–high correlations of Ptch1 transcript abundance with various genes indicative of observed features of Ptch^Hi^ Tmem (**Figures 5G–I**). We observed significant correlations involving transcript abundances of Ptch1 with cytokine effectors (**Figure 5G**, IFN-G, IL-17, IL-21), costimulatory molecules (**Figure 5H**, ICOS, PDCD1), and molecules conferring homing (**Figure 5I**, left, CCR2, CCR4) and retention (**Figure 5I**, right, CD103, S1PR5) within peripherally inflamed tissues.

### Ptch^Lo^ subsets preferentially home to lymphoid tissues *in vivo*

**Figure 6:**
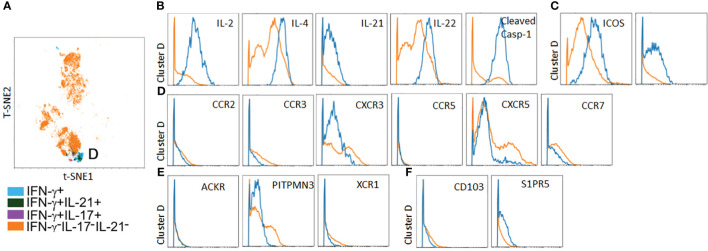
A polyfunctional Ptch^Lo^ subset preferentially home to lymphoid tissues *in vivo*. Ptch^Lo^ Tmem recovered from autologous LN tissues 5 days post-transfer were analyzed *ex vivo* via mass cytometry. Concatenated t-SNE plots from *n* = 3 separate donors identified one salient effector subset, cluster D **(A)**. Cluster D Tmem coexpresses effector cytokines **(B)** and costimulatory markers **(C)** but shows low expression of chemokine receptors **(D)** and atypical chemokine receptors **(E)**, tissue retention molecules **(F)** as well as upregulated expression of CXCR5 **(D)**.

In parallel to the above, we analyzed spatial positioning molecules on Ptch^Lo^ Tmem, which preferentially homed to autologous LN tissues. Following costimulation with αCD28 Ab Ptch^Lo^ Tmem labeled with IVIS770 dye, these were passively transferred into hosts bearing autologous LN and allogeneic, IRI-treated skin tissues. Ptch^Lo^ Tmem were recovered from LN tissues 5 days post-transfer and analyzed via mass cytometry (**Figure 6**). We found that, in contrast to Ptch^Hi^ Tmem, Ptch^Lo^ Tmem localized to autologous LN tissues showed only one salient, cytokine-producing cluster, which we termed cluster D (**Figure 6A**). Cluster D Tmem showed polyfunctional cytokine responses including IL-2, IL-4, IL-21, IL-22, and cleaved casp-1, whose activity generates IL-1β (**Figure 6B**). Cluster D Tmem also showed increased expression of costimulatory molecules (**Figure 6C**). In contrast to cluster A Tmem, Tmem in cluster D showed a distinct pattern of expression of spatial positioning molecules, showing relatively lower expression of positioning molecules conferring peripheral tissue homing including decreased typical chemokine receptors (CXCR3, CCR2, and CCR5, **Figure 6D**), atypical chemokine receptors (ACKR, PITNP3, XCR1, **Figure 6E**), and tissue retention molecules (CD103, S1PR5, **Figure 6F**) when compared to non-cytokine producing PtchLo Tmem. Ptch^Lo^ Tmem lacking cytokine expression showed increased expression of CCR7 and CXCR5, allowing lymphoid homing (**Figure 6D**). Together, these data indicate that Tmem shows markedly divergent cytokine effector responses and homing patterns *in vivo* based on the presence or absence of Hh costimulation.

## Discussion

In this study, we use multi-omic approaches in conjunction with a novel, humanized mouse model to resolve functional heterogeneity among a pathogenic Ptch^Hi^ T-cell population implicated in IRI. Human ECs release Hh morphogens following IRI, and these same ligands deliver potent costimulatory signals to heighten the effector and migratory responses of Ptch^Hi^ Tmem. In this study, we found that Hh costimulation induced oligoclonal and polyclonal expansion of Ptch^Hi^ Tmem, and these Hh-responsive clones showed highly polyfunctional cytokine but not chemokine profiles typified by markedly increased IFN-γ. Relative to Ptch^Lo^ Tmem receiving costimulation by CD28, Ptch^Hi^ Tmem differentially expressed molecules favoring homing to peripheral tissues and accordingly showed enhanced infiltration into IRI-treated human skin xenografts. Informed by multiplexed laser bead assays, single-cell proteomics, *and* mass cytometry, we visualized three clusters of Ptch^Hi^ Tmem that recapitulated effector features observed *in vitro*, including enhanced polyfunctionality and expression of positioning molecules, allowing homing to IRI-treated peripheral tissues. Our studies resolve heterogeneity within Ptch^Hi^ Tmem, an alloimmune T-cell subset mediating IRI-associated tissue injury.

We used human LNs as prototypical lymphoid tissues due to the availability of tissue samples and their high rates of surgical engraftment. A limitation on the use of these tissues is that, while becoming vascularized, implanted LNs do not form lymphatic drainage channels from peripheral skin tissues and thus do not reflect “draining” LNs. We found that human spleen implants elicited qualitatively similar homing with preferential recruitment of Ptch^Lo^ Tmem, and this occurred under similar kinetics. These observations suggested that LN tissues functionally recapitulate hematogenous trafficking of immune cells, similar to spleen tissues. Another limitation of our model is the inability to distinguish immune effects between major, i.e., MHC I/II, and minor alloantigens. In our system, skin and LN tissues are obtained from different donors to induce full MHC mismatch(es), the strength of which likely far outweighs immune responses induced by minor alloantigens like H-Y or other polymorphic alleles such as MICA.

Analyses of Ptch^Hi^ Tmem within inflamed skin tissues uncovered a subset, cluster 1, which demonstrated features of T_PH_ cells (**Figure 5**). This population, variably termed T follicular helper-like cells, expresses costimulatory molecules including ICOS, IL-21, and homing markers including CCR2/CCR5, but not CXCR5. Based on these phenotypes, T_PH_ cells are postulated to support Ab formation within peripheral tissues (35). We previously showed that IRI-treated ECs expanded T_PH_ cells in a human artery xenograft model (14) and that these cells could support the production of isotype-switched IgG binding to donor tissues. Our current multi-omics approaches confirmed that Hh costimulation expands T_PH_ cells, thus placing T_PH_ cells as a subset contained within Ptch^Hi^ Tmem. T_PH_ cells are expanded in numerous inflammatory conditions and may be similarly expanded via tissue-derived Hh costimulation, and this premise is yet to be explored.

Via differential approaches including mAb production (40) and limiting dilution (41), other groups have demonstrated that B cells contained within human donor tissues may produce Abs directed against antigens on donor tissues. As our prior humanized model lacked human lymphoid tissues, our current humanized model may enable focused interrogation as to how the Ptch^Hi^ population containing T_PH_ cells might contribute to alloAb responses.

The immune mechanism(s) underlying IRI of skin tissues are highly relevant to clinical settings involving extensive trauma and burns where skin allografts are frequently employed. While vascular composite allografts (VCAs) involving facial skin tissues are newly utilized and currently constitute a minority of transplanted tissues (42), our studies may show relevance to the rejection of these tissues (43), the immune mechanisms of which are becoming increasingly investigated. Ptch^Hi^ Tmem was newly described by our group (14) and as such, there are no antecedent reports in the literature describing its roles in VCAs. However, tissue-infiltrating subsets including Ptch^Hi^ Tmem (14), T_PH_ cells (35), and a recently described granzyme K^+^ T-cell population (44) show surface phenotypes compatible with enhanced peripheral tissue homing, and we surmise that one or more of these Tmem subsets may principally mediate VCA rejection.

While toleragenic strategies have been proposed, including CAR T and autologous Treg therapies (37, 43, 45), targeted therapies toward Tmem showing enhanced peripheral tissue homing, inclusive of Ptch^Hi^ Tmem, have not been explored in the setting of VCAs. Blockade of EC-mediated anti-VCAM1 interactions via biologics, including natalizumab and vedolizumab, has proven effective in ameliorating T-cell homing to inflamed tissues in autoimmune disease and may show promise in blocking immune complications attendant to VCAs.

## Data availability statement

The datasets presented in this study can be found in online repositories. The names of the repository/repositories and accession number(s) can be found below: GSE147089, GSE112943, GSE97779 (GEO).

## Ethics statement

The studies involving humans were approved by Yale Institutional Review Board. The studies were conducted in accordance with the local legislation and institutional requirements. Written informed consent for participation was not required from the participants or the participants’ legal guardians/next of kin in accordance with the national legislation and institutional requirements. The animal study was approved by Yale Institutional Animal Care & Use Committee. The study was conducted in accordance with the local legislation and institutional requirements.

## Author contributions

SW, QW, and DJ-W performed all *in vitro* experiments, *in vivo* experiments, and data analysis. GS, QJ, MN, and MF drafted the manuscript. JP and GT proofread the manuscript. ZT contributed to the methodology. DJ-W designed all of the experiments. All authors contributed to the article and approved the submitted version.
