# Supplementary material for: Hedgehog costimulation during ischemia-reperfusion injury potentiates cytokine and homing responses of CD4+ T cells
Source: Front Immunol. 2023 Oct 17;14:1248027. doi: 10.3389/fimmu.2023.1248027 (PMC10616247; doi:10.3389/fimmu.2023.1248027)
Supplement: Supplementary file 1 [file Presentation_1.pdf]

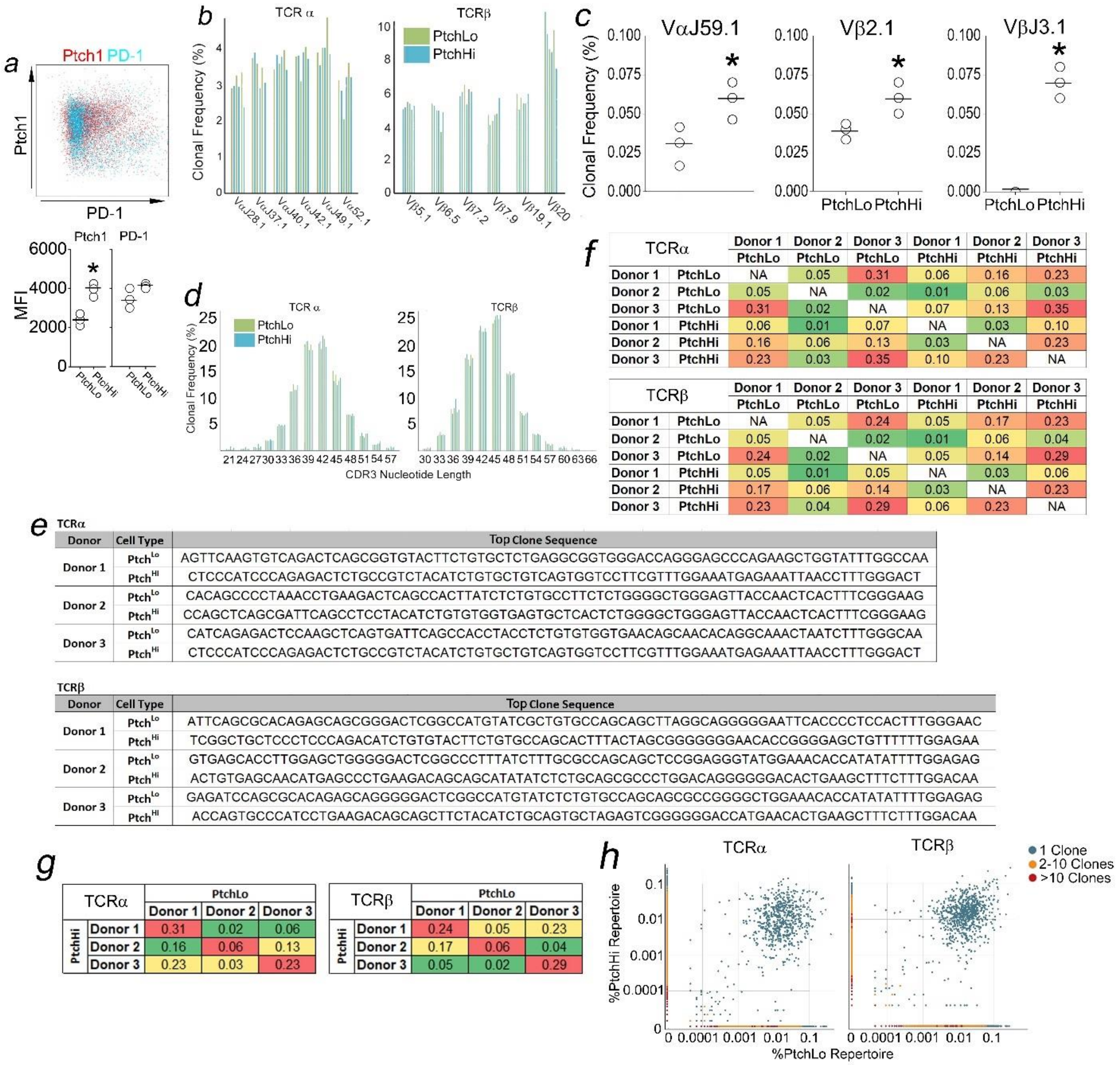

Figure S1. TCR Deep Sequencing of Hh-Costimulated Tmem. MFIs of Ptch1 and PD-1 among $Ptch^{Hi}$ and $Ptch^{Lo}$ Tmem gated on CD4+CD45RO+HLA-DR+ events (*a*). The most frequent V genes in TCRα and TCRβ did not significantly differ among $Ptch^{Lo}$ and $Ptch^{Hi}$ Tmem (*b*). Certain low frequency V genes showed significant differences among $Ptch^{Lo}$ and $Ptch^{Hi}$ Tmem (*c*). CDR3 nucleotide length did not significantly differ in TCRα and TCRβ among $Ptch^{Lo}$ and $Ptch^{Hi}$ Tmem (*d*). The top clones detected among TCRα and TCRβ rearrangements (*e*) showed low inter-donor similarity scores via Morista indices (*f*). Donors showed low similarity scores on a repertoire level as indicated by low Morista indices (*g*). Certain low-frequency clones were shared among all 3 donors (*h*). * indicates $p<0.05$. Student's *t*-test used for statistical comparisons in Fig S1*b*.

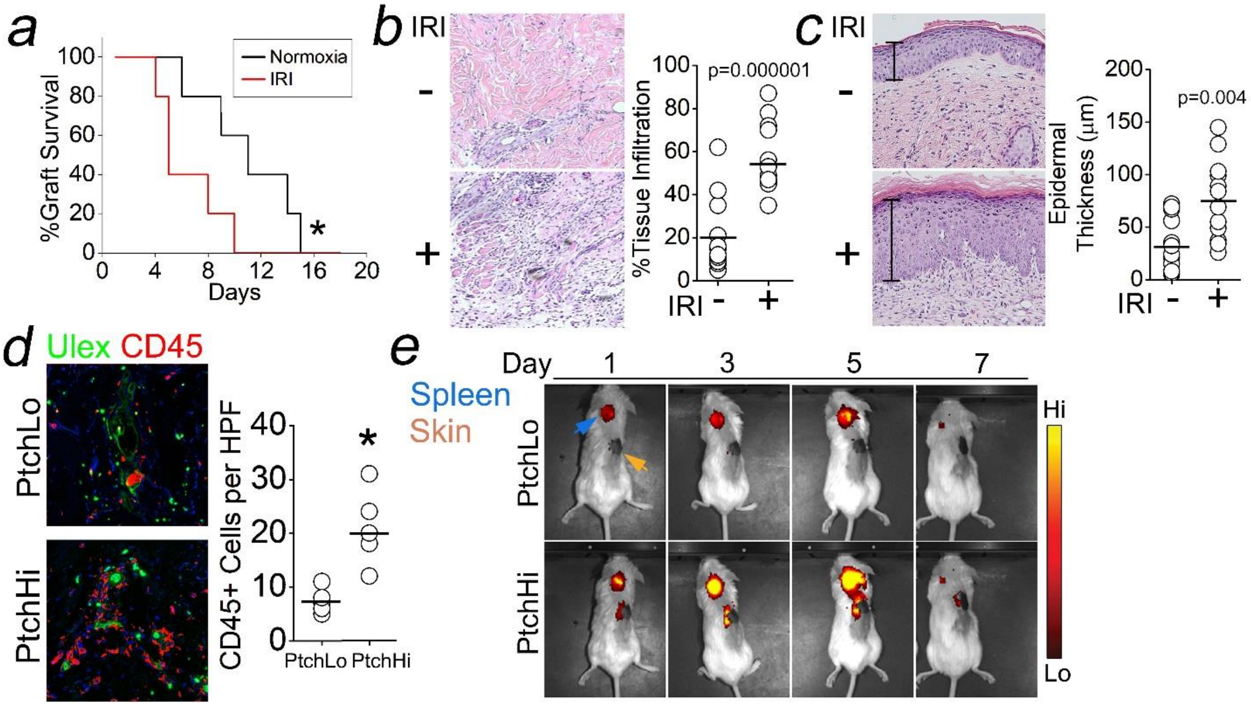

Figure S2. Effects of IRI on Human Skin and IVIS Imaging of Splenic Tissue Implants. Compared to skin subjected to normoxia, allogeneic human skin subjected to IRI showed worsened survival defined as >80% scab formation (*a*). Compared to controls, IRI-treated skin grafts showed increased immune cell infiltrates (*b*, n=5 per group) and significantly increased epidermal thickening (*c*) 14 days post-transfer. In hosts separately receiving either $Ptch^{Lo}$ or $Ptch^{Hi}$ Tmem, tissue-infiltrating human CD45+ cells were quantified in ≥5 hpfs per group 7 days post-transfer (*c*). Human spleen tissue and IRI-treated human skin were implanted into immunodeficient SCID/beige mice who passively received $Ptch^{Hi}$ or $Ptch^{Lo}$ Tmem that were autologous to spleen tissues and allogeneic to skin tissues. Migratory homing of passively transferred cells were imaged *ex vivo* over time (*e*). ≥3 hpfs per host were randomly selected and analyzed by 2 blinded reviewers (*b,c*). * indicates $p<0.05$ by Wilcoxon's rank sum test (*a*) or Student's *t*-test (*b*-*d*).

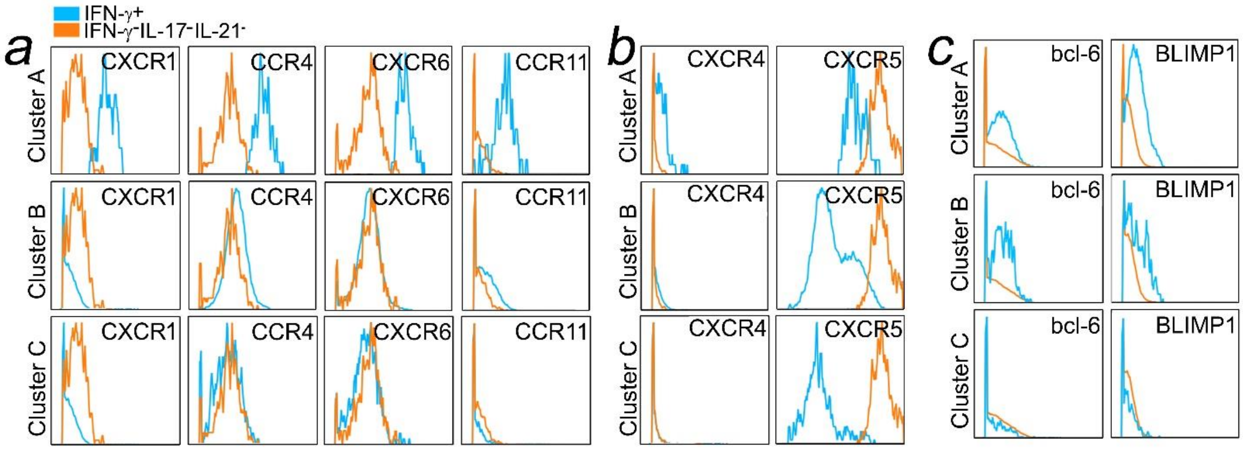

Figure S3. CyTOF Analyses of PtchHi Tmem in IRI-Treated Skin Tissues. Clusters A and C in $Ptch^{Hi}$ Tmem share expression of certain receptors and tissue retention molecules). Cluster A uniquely co-expresses a diversified panel of chemokine receptors annotated for homing to peripherally inflamed tissues (*a*) but not lymphoid organs (*b*). Cluster A Tmem show low bcl6:BLIMP1 ratios (*c*).
